# Enhanced Radar Signal Classification Using AMP and Visibility Graph for Multi-Signal Environments

**DOI:** 10.3390/s24237612

**Published:** 2024-11-28

**Authors:** Ji-Hyeon Kim, Soon-Young Kwon, Hyoung-Nam Kim

**Affiliations:** Department of Electrical and Electronics Engineering, Pusan National University, Busan 46241, Republic of Korea; kjihyeon@pusan.ac.kr (J.-H.K.); ysk1680@pusan.ac.kr (S.-Y.K.)

**Keywords:** radar scan pattern classification, amplitude pattern, visibility graph

## Abstract

Accurately classifying and deinterleaving overlapping radar signals presents a significant challenge in complex environments, such as electronic warfare. Traditional methods, such as spectrogram-based analysis, often struggle to differentiate radar signals with similar scan patterns, particularly under low signal-to-noise ratio (SNR) conditions. To address these limitations, we propose a novel two-stage classification framework that combines amplitude pattern (AMP) analysis and visibility graphs to enhance the accuracy and efficiency of radar signal classification. In the first stage, AMP analysis groups radar reception signals into broad categories, which reduces noise and isolates signal features. In the second stage, a visibility graph technique is applied to refine these classifications, enabling the practical separation of radar signals with overlapping or similar amplitude features. The proposed method is particularly effective in handling complex scans, such as the Palmer series, which blends search and tracking patterns. Deep learning models, including GoogLeNet and ResNet, are integrated within this framework to improve classification performance further, demonstrating robustness in low-SNR and multi-signal environments. This approach offers significant improvements over conventional methods, providing enhanced performance in differentiating radar signals across various scanning patterns in challenging multi-signal environments.

## 1. Introduction

In modern electronic warfare (EW) systems, the ability to detect, classify, and analyze enemy radar signals is crucial for gaining tactical advantages. As radar technology has evolved, so has the complexity of radar signal environments, where multiple overlapping radar signals from different sources are received simultaneously. An accurate distinction between these signals is critical for ensuring effective signal processing and providing real-time situational awareness. Radar scan patterns are key indicators that provide valuable insights into the type, purpose, and operational status of enemy radar systems, making them essential for effective signal classification.

Traditional radar identification methods rely on signal parameters, such as frequency, pulse width, and pulse repetition interval (PRI), to classify radar types [1,2,3]. However, the rapid increase in electronic equipment and the development of advanced radar jamming and countermeasures have degraded the electromagnetic spectrum environment. This has led to ambiguities in signal identification because these conventional parameters alone are often insufficient to distinguish between radar types, particularly when signals share similar characteristics. Consequently, there is growing interest in utilizing additional radar signal attributes, such as scan patterns, to improve the classification performance [4,5,6,7,8,9,10].

Radar scan patterns, such as circular, sector, raster, and helical patterns for search radar, and conical and lobe switching for tracking radar, provide essential information for identifying radar types and its operational modes. In more complex scenarios, hybrid scan patterns, such as Palmer scans, which are combinations of search and tracking patterns, are often encountered. These scan patterns offer insights into the strategic intentions of adversarial radar systems, thereby enabling more informed defense and offense strategies.

Conventional radar scan pattern classification methods [5,6,7], such as those based on spectrogram analysis or peak feature extraction, often struggle with signal-to-noise ratio (SNR) limitations or with similar radar patterns. To overcome these challenges, recent studies [4,11] have explored additional features for scan pattern classification for more effective scan pattern classification in complex environments. However, these approaches primarily focus on simple classification tasks and often face challenges in handling diverse or overlapping scan patterns encountered in multi-signal environments including the complex Palmer series patterns.

In this article, we propose a novel radar scan pattern classification algorithm that models radar reception signals based on various operational conditions and extracts critical features through a two-stage approach: amplitude pattern (AMP) analysis [11] and visibility graph techniques [5]. The two-stage approach is designed to sequentially address the complexities of radar signal classification by initially simplifying the classification problem and then focusing on detailed structural analysis.

In the first stage, the AMP-based method is applied to perform a coarse classification, grouping signals into broad categories (e.g., conical, search, Palmer series, and lobe-switching patterns). The AMP captures variations in the signal amplitude over time, which directly correspond to different radar scan behaviors. This initial grouping serves as a noise reduction mechanism by isolating patterns from broader classes, thereby minimizing the impact of background noise or minor fluctuations. By processing a simplified and organized set of grouped signals, the visibility graph analysis in the second stage can focus on capturing subtle structural patterns critical for detailed classification, even in challenging low-SNR environments.

Following the AMP-based classification, the visibility graph method is employed to extract critical features from radar signals by transforming time-series data into a network representation. This graph-based approach is highly effective for capturing both structural and dynamic changes in radar signals, enabling precise classification even in environments with similar radar scan patterns.

To enhance classification performance, deep learning algorithms, such as GoogLeNet [12] and ResNet [13,14], are integrated as auxiliary tools within the AMP and visibility graph framework. While various deep learning models could be used, GoogLeNet and ResNet were chosen specifically for their architectural strengths: GoogLeNet’s multiscale feature extraction and ResNet’s resilience to performance degradation in deeper networks. This integration with deep learning enhances the effectiveness of visibility graph-based classification while keeping the focus on the contributions of the AMP and visibility graph framework. Instead of relying solely on deep learning, this study leverages the complementary strengths of AMP analysis and visibility graphs within a two-stage structure to create a robust radar signal classification framework. This approach is especially suited for radar environments with overlapping signals and low SNR, offering a novel approach that goes beyond the limitations of conventional deep learning or spectral-based methods.

The remainder of this paper is organized as follows: Section 2 describes the signal modeling techniques and amplitude representation used to generate the radar reception signals for different scan patterns. Section 3 introduces the proposed radar scan pattern classification algorithm, including the decision tree structure and visibility-graph-based refinement. Section 4 presents the application of deep learning models for the classification of radar signals. Section 5 presents the experimental results. Finally, Section 6 concludes the paper and presents future research directions.

## 2. Signal Modeling and Amplitude Pattern Representation

### 2.1. Received Radar Signal Modeling [6,7]

Received radar signal modeling is critical for understanding how radar signals are processed in an electronic warfare support (ES) system. The modeling technique consists of a part that determines the pulse arrival time, which is the moment at which the radar reception signal is received by the ES system; a part that determines the received signal strength; and a part that reflects the noise that occurs in an actual EW environment.

When the radar reception signal is collected from $t_{1}$ to $t_{N}$, the n-th pulse arrival time $t_{n}$ can be expressed by the pulse interval ${p(t}_{n})$ determined by PRI modulation:

(1) $$t_{n}=t_{n-1}+{p(t}_{n}),n=1,\cdots ,N.$$

**Figure 1:**
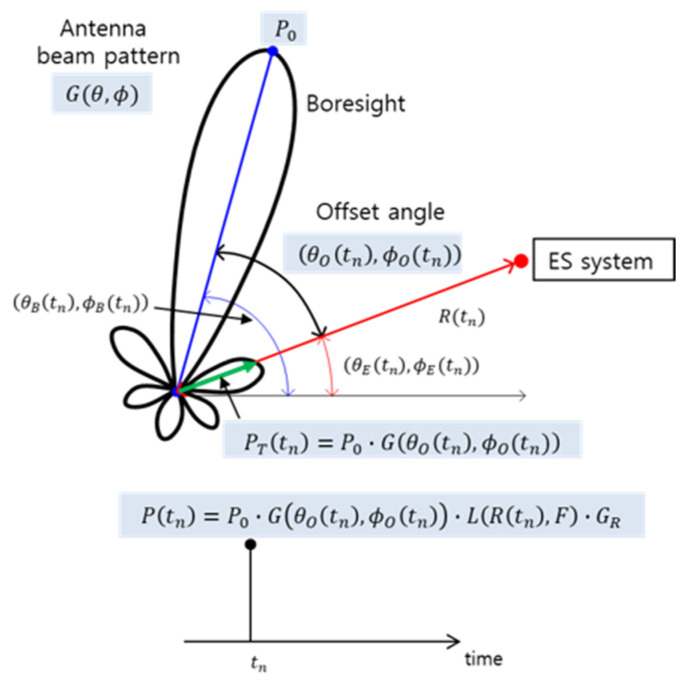
Model of the received radar signal strength [6].

The received signal strength at each pulse arrival time is primarily influenced by the radar’s scanning pattern, transmission power, and environmental factors, such as distance, antenna gains, and free-space path loss [6]. As shown in the radar received signal strength model in Figure 1, the offset angle between the radar boresight and the ES system at a specific time $t_{n}$ plays a significant role. The offset angle $\theta_{O}(t_{n})$ and $\phi_{O}(t_{n})$ is determined as the angular difference between the ES system’s direction of $\theta_{E}(t_{n})$ and $\phi_{E}(t_{n})$ and the radar’s boresight of $\theta_{B}(t_{n})$ and $\phi_{B}(t_{n})$. The transmission power $P_{T}(t_{n})$ directed toward the ES system is described as a function of the maximum transmission power $P_{0}$ and the gain $G(\theta_{O}\left(t_{n}\right),\phi_{O}\left(t_{n}\right))$ dependent on the offset angle:

(2) $$P_{T}\left(t_{n}\right)=P_{0}\cdot \;G(\theta_{O}\left(t_{n}\right),\phi_{O}\left(t_{n}\right)).$$

This transmitted signal experiences free-space propagation loss as it travels toward the ES system, resulting in a received signal power $P(t_{n})$:

(3) $$P(t_{n})=P_{T}\left(t_{n}\right)\cdot \;L(R\left(t_{n}\right),F)\cdot \;G_{R},$$

where $L(R\left(t_{n}\right),F)$ represents the free-space path loss depending on the distance $R\left(t_{n}\right)$ and frequency F, and $G_{R}$ is the receiving antenna gain.

In addition, noise is added to simulate realistic conditions; the SNR is calculated for each pulse, and additive white Gaussian noise is incorporated.

These parameters constitute the received radar signal model, which effectively simulates the radar signal characteristics under various scan patterns and operating environments.

### 2.2. Amplitude Pattern Representation [10,11]

**Figure 2:**
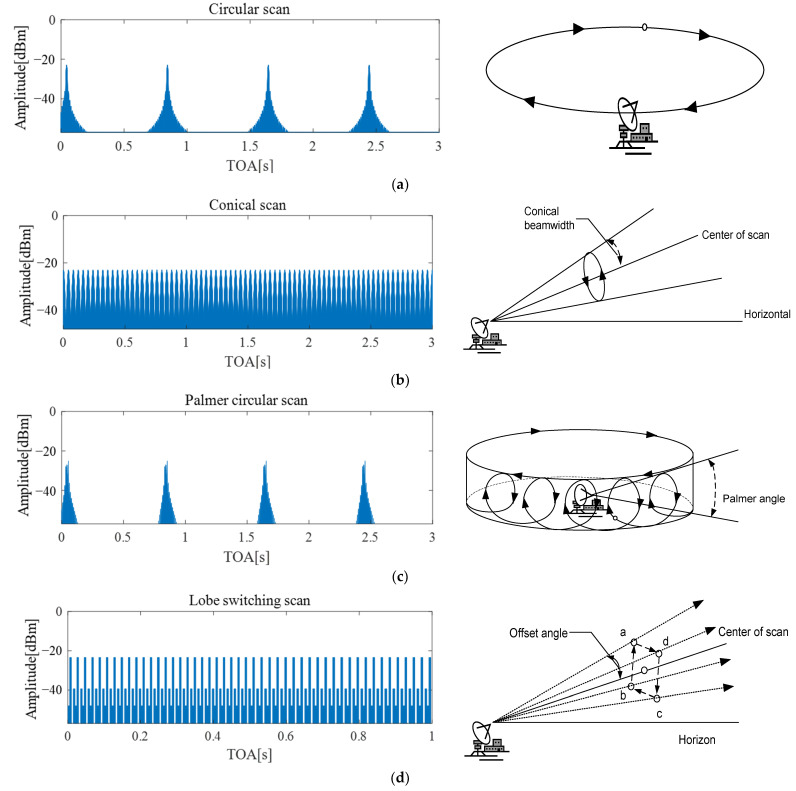
AMPs with different radar antenna scan types: (**a**) circular scan; (**b**) conical scan; (**c**) Palmer circular scan; (**d**) lobe-switching scan.

According to the amplitude patterns (AMPs) of signals observed in electronic intelligence (ELINT) systems, search and tracking radars exhibit different behaviors, as shown in Figure 2. For search radars, such as circular, sector, raster, and helical scan types, the AMP typically appears as a sinc function owing to the movement of the radar beam. In the case of tracking radars, such as lobe-switching, electronic lobe-switching, and conical scan types, the AMP at the receiver is usually observed as either a constant or a sinusoidal pattern. By extension, for the Palmer scan radar signals, which are hybrids of search and conical tracking radars, the AMP of the receiver is typically a combination of sinc and sinusoidal patterns. The unique combination of the circular, sector, and conical scan characteristics of the Palmer scan leads to more complex amplitude variations that integrate the features of search and tracking radar patterns.

The sinc AMPs [11] caused by circular, sector, raster, and helical scan radars are given by

(4) $$AMP_{n}=S\left({TOA}_{n}\right)+\left|C\frac{\sin\left(\frac{2\pi TOA_{n}}{T}\right)}{\frac{2\pi TOA_{n}}{T}}\right|-C+W_{n},$$

where $S\left({TOA}_{n}\right)$ represents the offset value indicating the maximum amplitude value in the pattern, and $W_{n}$ represents Gaussian measurement noise with a mean of zero and a standard deviation of σ. C is a constant used to control the sidelobe level of the sinc pattern, and T refers to the illumination time. The absolute value notation $\left|\cdot \right|$ denotes the amplitude adjustment for sinc modulation, which ensures non-negative values in the AMP for accurate radar scan pattern modeling.

The sinusoidal AMP caused by the conical scan [11] is formulated as follows:

(5) $$AMP_{n}=S\left({TOA}_{n}\right)+D\sin\left(\frac{2\pi TOA_{n}}{P}+\phi \right)+W_{n},$$

where P represents the period and D denotes the peak value of the pattern. The phase of the sinusoid ϕ is assumed to be uniformly distributed in the interval [0,2π).

In contrast, more complex scanning techniques like the Palmer series exhibit additional modulations in their AMPs. For instance, a Palmer circular scan combines conical and circular scan motions, resulting in an AMP that integrates a sinc function with sinusoidal variation. This hybrid form of modulation requires a more nuanced mathematical representation to capture the combined effects of the two scanning methods.

To address the unique characteristics of Palmer series scans, we derived a new equation, as shown in (6), specifically to model the AMPs observed in these hybrid scanning motions. This derived equation effectively captures the complex interaction between the sinusoidal modulation characteristic of conical scans and the sinc modulation seen in other patterns within the Palmer series. The derived AMP equation has the following hybrid form:

(6) $$AMP_{n}=S\left({TOA}_{n}\right)+D\sin\left(\frac{2\pi TOA_{n}}{P}+\phi \right)\cdot \left|C\frac{\sin\left(\frac{2\pi TOA_{n}}{T}\right)}{\frac{2\pi TOA_{n}}{T}}\right|+W_{n}.$$

On the other hand, the constant AMP observed in the ELINT system can be modeled as follows [11]:

(7) $$AMP_{n}=S\left({TOA}_{n}\right)+W_{n},$$

where $AMP_{n}$ and ${TOA}_{n}$ represent the amplitude and time of arrival (TOA) of the n-th pulse, respectively. The total number of pulses is denoted by N, which has the maximum value of n. In this model, $S\left({TOA}_{n}\right)$ remains constant for the lobe-switching and electronic lobe-switching scan radar types.

## 3. Tree-Structured Scan Pattern Classification Method

In this section, we propose a decision-tree-based algorithm for effectively classifying radar scan patterns from the signals received by an ES system. This approach categorizes the received signals according to the 11 representative scan patterns modeled in Section 2 through a hierarchical structure that refines classifications progressively. The algorithm initiates with a general categorization using AMP analysis at the top node and then narrows down to specific patterns in subsequent nodes through a visibility graph. This hierarchical structure reduces computational complexity by first organizing signals into broad categories, allowing visibility graph analysis to focus on detailed classification within each category.

**Figure 3:**
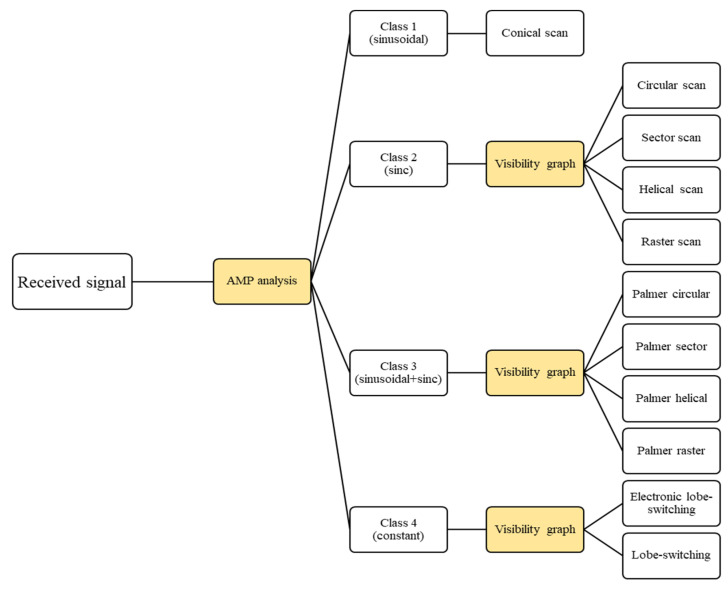
Tree-structured radar scan pattern classification algorithm.

The proposed algorithm operates in two distinct stages, as shown in Figure 3. In the first stage, the AMP analysis categorizes radar signals into broader types, such as conical, circular, sector, raster, helical, Palmer, and switching scan patterns. In the second stage, a visibility-graph-based method is applied to further differentiate between the subclasses within each main category. This two-step approach enhances classification accuracy by combining amplitude variations (in AMP) and temporal relationships (in visibility graphs). This is critical in effectively deinterleaving radar signals, especially in low-SNR and multi-signal environments.

### 3.1. Stage 1: Initial Classification Using AMP

The first stage leverages amplitude pattern analysis for broad categorization, grouping radar signals into categories based on their characteristic amplitude modulations. The AMP-based grouping reduces background noise and highlights primary signal features, making it easier to capture subtle differences in complex patterns later in the classification process. This process effectively organizes the radar signals into four distinct classes: sinusoidal, sinc, sinusoidal+sinc, and constant, as listed in Table 1. Each class encompasses various radar scan patterns, with sinusoidal corresponding to conical scans; sinc covering circular, sector, raster, and helical scans; sinusoidal+sinc representing Palmer scan patterns; and constant referring to electronic lobe-switching and lobe-switching radar signals.

**Figure 4:**
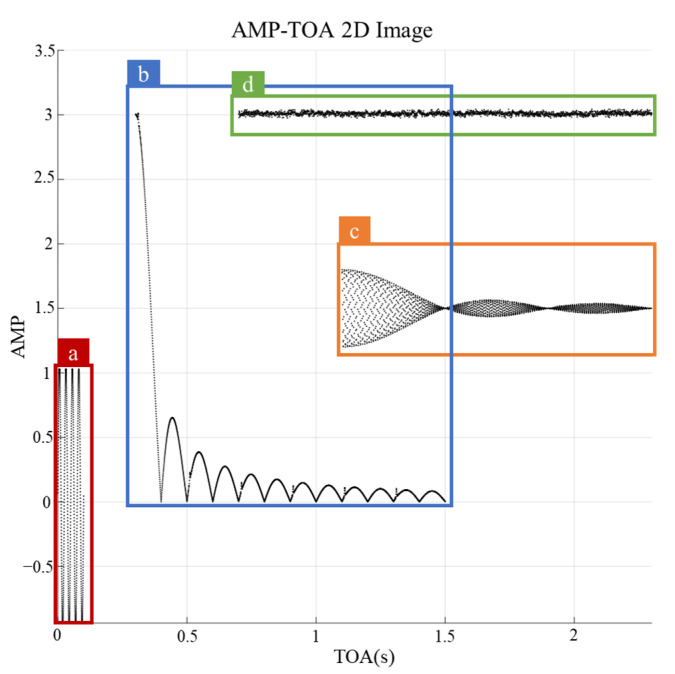
Scan type identification results for a generated AMP-TOA image containing patterns of four different classes: (**a**) sinusoidal; (**b**) sinc; (**c**) sinusoidal+sinc; (**d**) constant.

Figure 4 illustrates the AMPs for the representative signals in each of the four classes: sinusoidal (conical scan), sinc (circular scan), sinusoidal+sinc (Palmer circular scan), and constant (lobe-switching). The AMP-based classification method relies on plotting the amplitude of the radar reception signal against its TOA, allowing the unique features of each radar scan pattern to emerge.

- Sinusoidal patterns (Class a): These patterns are typically associated with conical scans, and result in sinusoidal waveforms in the AMP plot. Owing to the circular motion of the radar beam, the amplitude repeatedly increases and decreases, producing a periodic sinusoidal curve, as shown in Figure 4. This oscillation reflects a continuous beam rotation, making it a distinctive identifier for conical scans.
- Sinc patterns (Class b): The signals from circular, sector, raster, and helical scans belong to this category. The AMP plot for these scans displays a sinc-like waveform, characterized by a series of sidelobes formed as the radar beam sweeps across wide areas. The strength of the received signal fluctuates, creating a periodic ripple effect in the amplitude. This is a critical feature for identifying these radar types.
- Sinusoidal+Sinc patterns (Class c): Palmer scans, which combine the characteristics of both sinusoidal and sinc patterns, present more complex waveforms. In this class, the main and side lobes overlap in the AMP plot, reflecting the intricate movement of the radar beam. Palmer circular, sector, raster, and helical scans generate these intertwined patterns, as shown in Figure 4.
- Constant patterns (Class d): Radar signals obtained using electronic lobe-switching or lobe-switching techniques exhibit relatively constant AMPs over time. These patterns are primarily flat in the AMP plot, with occasional small deviations caused by switching events. The near-constant behavior in these plots enables an easy distinction from the more dynamic waveforms of the other classes.

This broad categorization through AMP serves as an effective pre-processing step, reducing background noise by filtering out minor fluctuations and focusing on core structural features, which is particularly useful in low-SNR environments. This first-stage classification provides a robust foundation for the more refined analysis in Stage 2, leveraging the structural characteristics captured by AMP.

### 3.2. Stage 2: Visibility-Graph-Based Detailed Classification

Visibility graphs [5] convert time-series data into networks and are widely used in various fields, such as time-series analysis and pattern recognition. In a visibility graph, each data point in the time series is considered a node, and nodes are connected if they have ‘visibility’ with each other. This allows structural characteristics and dynamic changes in the time series to be captured and represented as networks. This network representation enables the algorithm to capture both the structural characteristics and dynamic changes in the radar signal’s time-series data, making it particularly suited for distinguishing similar radar scan patterns with overlapping characteristics.

If the amplitude of any three points in a radar reception signal containing scan pattern information is expressed as coordinates, it can be assumed as $\left(t_{\alpha },A_{\alpha }\right),\left(t_{\beta },A_{\beta }\right),\;\left(t_{\gamma },A_{\gamma }\right)$. If there is visibility between two points, α and β, the nodes can be connected in the graph. If there is a third point γ between the two points, the condition of (8) must be satisfied.

(8) $$A_{\gamma }<A_{\alpha }+\left(\frac{{A_{\beta }-A}_{\alpha }}{{t_{\beta }-t}_{\alpha }}\right)\cdot \left({t_{\gamma }-t}_{\alpha }\right).$$

This condition indicates that γ is not on the straight line between α and β; hence, γ does not interfere with the visibility between α and β. The computational complexity of generating a visibility graph is generally $O(N^{2})$, where N represents the number of data points in the radar signal. This complexity arises from the need to compare each node with every other node to establish visibility.

**Figure 5:**
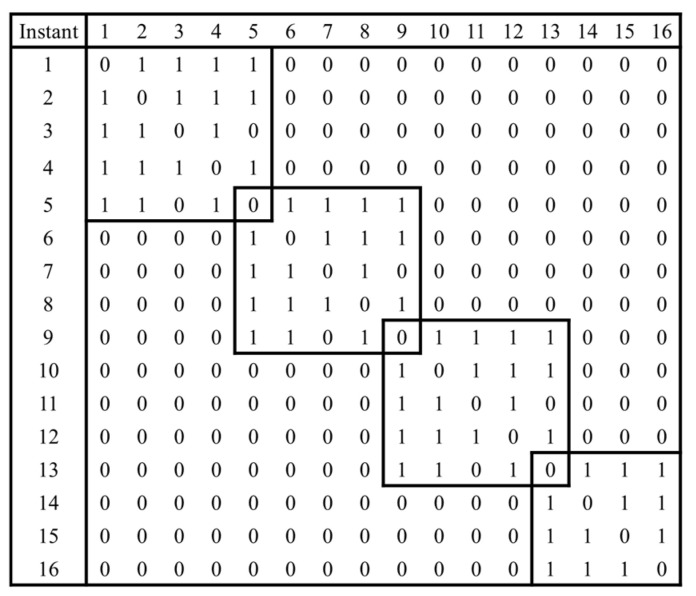
The degree distribution matrix of the visibility graph [5].

When converting a time series into a visibility graph in this way, we can define a degree distribution matrix, which is the number of connections between the nodes in the visibility graph. If there is a connection between α and β, the columns and rows of the visibility graph matrix have a value of 1; otherwise, they have a value of 0. An example of a visibility graph matrix constructed according to this rule is shown in Figure 5. The degree distribution matrix, which varies with different radar scan patterns, enables effective differentiation of scan patterns by highlighting distinct structural features.

**Figure 6:**
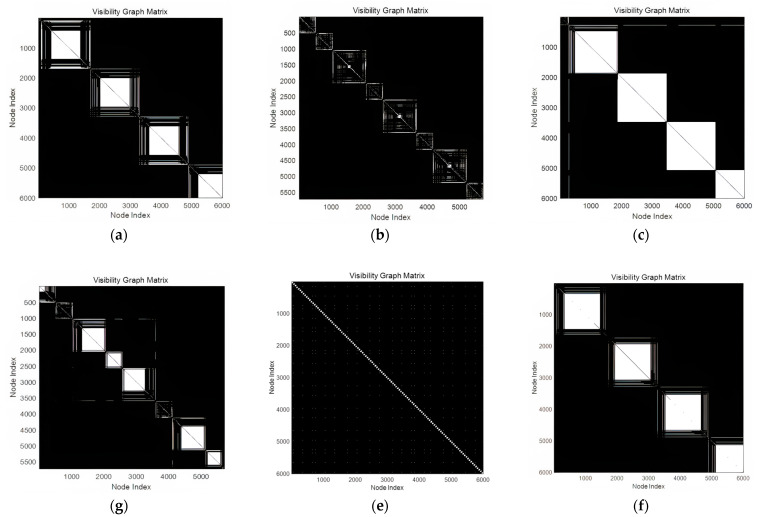

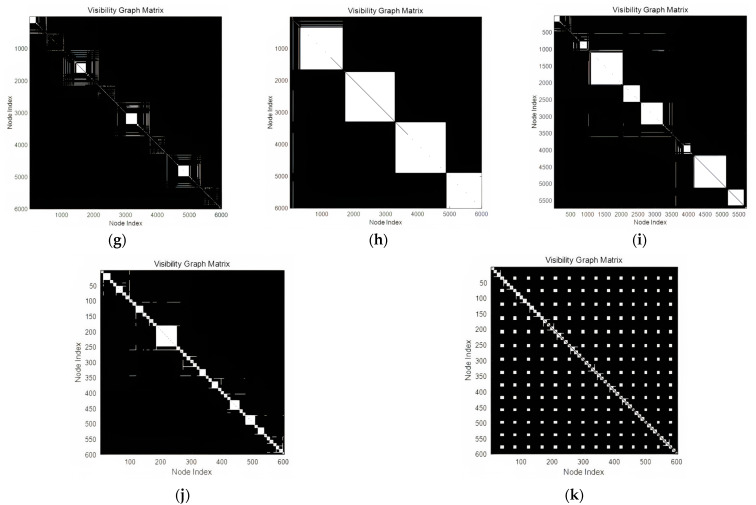
Visibility graph of the radar scan pattern received signals: (**a**) circular scan; (**b**) sector scan; (**c**) 4-bar helical scan; (**d**) 4-bar raster scan; (**e**) conical scan; (**f**) Palmer circular scan; (**g**) Palmer sector scan; (**h**) 4-bar Palmer helical scan; (**i**) 4-bar Palmer raster scan; (**j**) electronic lobe-switching; (**k**) lobe-switching.

Figure 6 shows the visibility graphs corresponding to the radar reception signals for the various scan methods. The circular scans, as depicted in the visibility graph, exhibited a repetitive pattern owing to the uniform motion of the radar beam, which consistently scanned a 360° azimuth. This uniform motion causes the signal strength to remain consistent over time, resulting in repeated structures in the visibility graph. In contrast, the bidirectional sector scans displayed smaller patterns with two distinct shapes alternating in the visibility graph. The bidirectional movement of a radar beam generates alternating AMPs that manifest as distinct repetitive structures. The helical scans produced visibility graphs similar to the circular scans, but with variations in pattern size owing to the increasing elevation angle during scanning. As the elevation angle increased, the primary lobes in the signal became smaller, leading to visible connections between the larger and smaller lobes in the graph. The raster scans, which also utilize a multi-bar scanning structure, exhibited smaller primary lobes at higher elevations, forming connections between lobes of different sizes in the visibility graph. The conical scans that follow a sinusoidal pattern created distinct diagonal repetitions in the visibility graph. In contrast to the other scan patterns, a periodic sinusoidal signal generated specific connection points that appeared regularly in the graph. The Palmer scans, which combine conical with circular, sectoral, raster, and helical scans, exhibited unique high-frequency components in their visibility graphs. The Palmer circular and sector scans resembled the visibility graphs of the corresponding non-Palmer scans, but included areas where the high-frequency components disrupted visibility. The Palmer helical and raster scans also shared similarities with their respective standard versions; however, the high-frequency distortions inherent in the Palmer scans caused noticeable differences in the visibility graphs.

In addition, electronic lobe-switching and lobe-switching scans produced distinct visibility graph structures. The electronic lobe-switching scan generated a visibility graph with periodic patterns interrupted by switching events. By contrast, the lobe-switching scan created a more structured diagonal pattern owing to the regular positioning of the beam.

The visibility graph reflects both global and local patterns in the time-series data, which enables the analysis of the dynamic changes in the data. Therefore, the primary objective of this study is to use the visibility graph results for the received signals according to the scanning radar as input data for a deep learning algorithm. This approach allowed us to effectively classify the types of scanning radars, thereby contributing to the fields of radar technology and signal processing.

## 4. Deep Neural Network Algorithms: GoogLeNet and ResNet

In this section, we examine the application of deep learning models, specifically GoogLeNet and ResNet, to enhance radar signal classification within our two-stage AMP and visibility graph framework. GoogLeNet and ResNet were strategically chosen for their robust capabilities in feature extraction. These deep learning models enhance the accuracy and adaptability of our classification algorithm, enabling effective analysis across varying radar scan patterns.

### 4.1. GoogLeNet [12]

**Figure 7:**
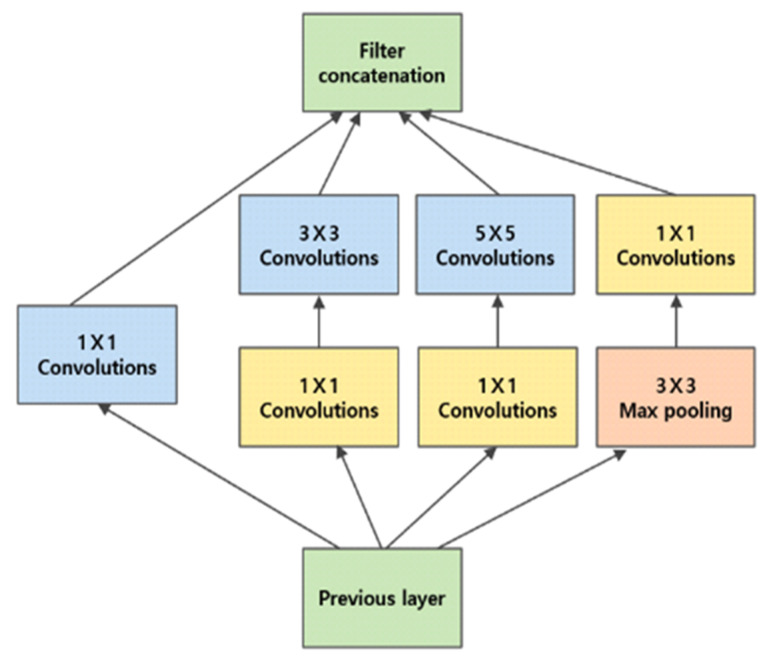
Inception module model of GoogLeNet [12].

GoogLeNet is a neural network architecture that won the 2014 ILSVRC (ImageNet Large-Scale Visual Recognition Challenge). The key innovation of this model lies in the introduction of the inception module, as shown in Figure 7, which enables both the expansion of the network depth and the broadening of its width. Traditional convolutional neural network (CNN) architectures use layers that are sequentially connected, each consisting of a single convolutional operation followed by pooling. GoogLeNet uses inception modules that apply parallel convolutional filters of varying sizes and pooling operations, and the results are concatenated along the channel dimensions. The inception module integrates 1 × 1, 3 × 3, and 5 × 5 convolutional filters with 3 × 3 max-pooling operations, thereby allowing the network to process information at different scales simultaneously. Among these, the 1 × 1 convolution plays a crucial role in reducing computational complexity and improving network efficiency.

GoogLeNet introduces two auxiliary classifiers positioned at the intermediate layers to address the vanishing gradient problem that often arises in deep networks. These auxiliary classifiers are used only during training, and help improve the flow of gradients, thereby facilitating better learning in the earlier layers of the network. Furthermore, rather than using fully connected layers, GoogLeNet adopts global average pooling in the final layer, which calculates the average of each feature map to generate a one-dimensional vector. This method significantly reduces the number of parameters and lowers the complexity of the model while maintaining its performance.

### 4.2. ResNet [13,14]

ResNet [13,14] is one of the most widely adopted CNN algorithms owing to its superior classification performance. The network consists of 3 × 3 repeated convolutional layers. ResNet has five variants based on the number of layers: ResNet18, ResNet34, ResNet50, ResNet101, and ResNet152. As the number of layers increases, the computational complexity and the number of parameters increase, as do the potential for higher accuracy and smaller test errors. The top five validation errors for the ImageNet dataset were approximately 5.6% for ResNet34, 5.25% for ResNet50, 4.6% for ResNet101, and 4.49% for ResNet152 [13]. When comparing ResNet34 and ResNet101, the latter demonstrated approximately 1% higher classification accuracy. In terms of computation, ResNet34 performs $3.6\times 10^{9}$ floating-point operations per second (FLOPs), whereas ResNet101 performs twice as many FLOPs, reaching 7$.6\times 10^{9}$ FLOPs [13]. Therefore, ResNet34 offers an optimal balance between the computational efficiency and the classification performance.

**Figure 8:**
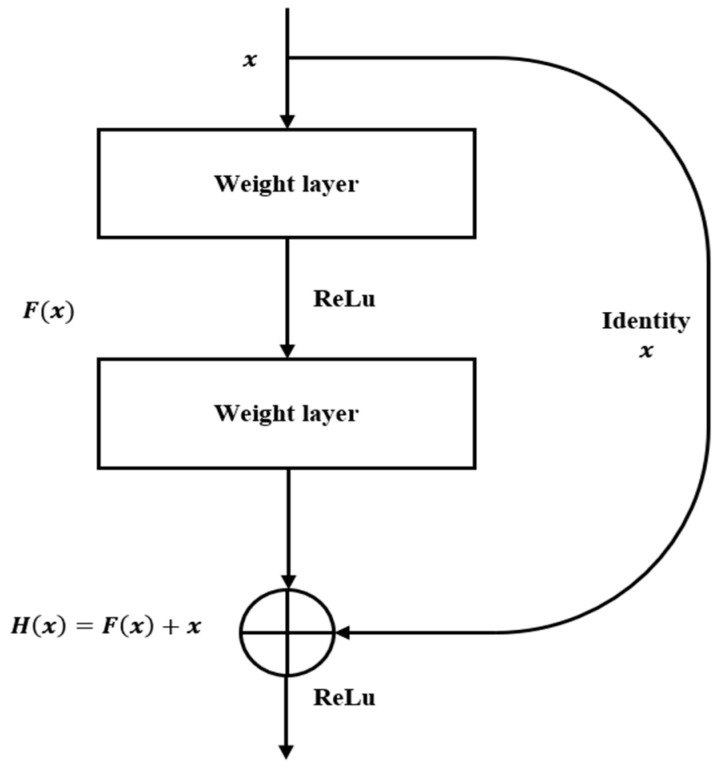
“Basic-block” building block for ResNet [13].

Figure 8 illustrates the shortcut connection structure in the ResNet algorithm, which is essential for its function. In the ResNet34 architecture, after passing through 16 shortcut structures, the algorithm can classify the input data with a high accuracy. These shortcuts, also referred to as identity mappings, allow the input to bypass unaltered convolutional layers. This preserves critical information while the data are processed through the network, ultimately improving classification outcomes. The convolution layers in the weight blocks handle feature extraction with a rectified linear unit activation function employed to prevent the performance degradation expected in deep networks. The objective of a traditional neural network is to approximate the output H(x) directly, but ResNet instead focuses on learning the residual function F(x)=H(x)−x. This residual learning approach simplifies the parameter optimization and mitigates issues, such as vanishing and exploding gradients. Identity mappings in the shortcut connections of ResNet contribute to improved classification accuracy while reducing network complexity.

## 5. Performance Evaluation

The following section presents the performance of the proposed radar scan pattern classification algorithm using GoogLeNet and ResNet34, as described in Section 4.

### 5.1. Experimental Environments

The radar reception signals used for classification were generated by considering various operational variables according to the 11 representative scan patterns based on the received radar signal modeling technique described in Section 2. The numbers of images used during training and testing were 2600 and 700, respectively. The size of the image was set to 600 × 600 pixels. The parameter values used to generate the training and test datasets and the intervals from which these values were chosen are presented in Table 2. A visibility graph based on the generated scan pattern signals was applied as the input data for deep learning. Furthermore, the Adam optimizer [12] was used for the weight-updating process, the number of epochs was 300, and the minibatch size was 64.

### 5.2. Performance Evaluation Metrics [15]

**Figure 9:**
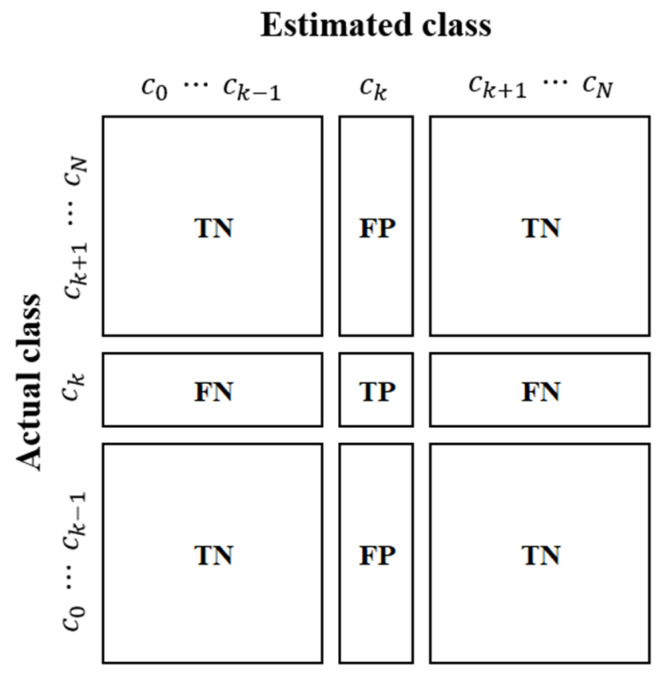
Confusion matrix for multi-class classification [15].

A confusion matrix is an N×N grid used to assess the performance of the classification model, where N denotes the number of target classes. This matrix allows a comparison between the actual target values and the predictions made by the model, providing an apparent visual representation of the accuracy and misclassification rates of the model. Figure 9 illustrates the confusion matrix for the multiclass classification model with N classes. The matrix compiles the correct and incorrect classifications for each class into a confusion matrix $C:=\left(c_{ij}\right)$, where $c_{ij}$ represents the number of instances in which the actual class i is estimated by class j. The actual class is the actual value, whereas the predicted class is the output of the model. TP and TN indicate instances where the model correctly predicted the actual values, whereas FP and FN represent instances where the predictions of the model differed from the actual values. Each term is defined as follows:

1. TP (true positive): accurate classification of the category of interest.
2. TN (true negative): accurate classification of items not in the category of interest.
3. FP (false positive): misclassification of noninterest categories as categories of interest.
4. FN (false negative): incorrect classification of a category of interest as not a category of interest.

Based on the aforementioned four components, the following three measures can be derived: accuracy, precision, and recall. Accuracy represents the proportion of correctly classified instances in the total dataset, reflecting the overall effectiveness of the classification model. Precision measures the proportion of TP predictions among the total number of predicted instances for a specific class, indicating the reliability of the model in identifying the correct category. Recall assesses how well the model captures all the relevant instances for the category of interest, comparing the number of correct predictions to the actual number of instances in that category. These metrics are calculated as follows:

(9) $$Accuracy=\frac{TP+TN}{TP+FP+TN+FN},$$

(10) $$Precision=\frac{TP}{TP+FP},$$

(11) $$Recall=\frac{TP}{TP+FN}.$$

The F1-score, which is a crucial metric in machine learning, is used to assess the predictive performance of the AI model for each class. It is calculated as the harmonic mean of the precision and recall, providing a balance between the two when they are equally important. The F1-score ranges from 0.0 to 1.0, with values closer to 1.0 reflecting better model performance. Generally, an F1-score of 0.7 or higher is deemed acceptable. However, the ideal threshold is not fixed and can vary significantly depending on the specific use case and requirements, demonstrating the adaptability of the F1-score. In scenarios in which the consequences of FPs and FNs are more critical, a higher F1-score is essential.

(12) $$F1-score=2\times \frac{Precision\times Recall}{Precision+Recall}$$

### 5.3. Classification Results

#### 5.3.1. Stage 1: AMP-Based Signal Class Categorization

The first stage of the proposed classification algorithm focuses on the AMP-based signal categorization, in which the radar reception signals are grouped into four main classes: sinusoidal, sinc, sinusoidal+sinc, and constant. These categories were differentiated based on the unique AMPs inherent to various radar scan types, as outlined in Table 1. This stage plays a vital role in providing an initial classification that lays the groundwork for more detailed signal categorization processes in later stages.

The classification method employed in this study closely follows the technique proposed in [11] for the object-detection-based deinterleaving of radar signals using deep learning. Both methods analyze the AMP and TOA data to classify radar signals. In the referenced study, the AMP and TOA data were transformed into 2D images, making the AMPs visually interpretable and facilitating accurate signal classification.

Similarly, in this study, the AMP-TOA images generated during the initial classification stage enabled the separation of the radar signals into distinct classes. The patterns created by the radar’s scanning motion were visualized in these images, which helped the model effectively distinguish between broader signal categories. A key distinction of this study is the successful inclusion of Palmer series scans (sinusoidal+sinc), which are particularly challenging to classify because of their hybrid sinusoidal and sinc characteristics. By accurately categorizing these complex patterns, our approach extends the AMP-based method beyond previous studies and offers a more comprehensive and reliable solution for the classification of radar signals.

Table 3 presents the classification accuracies achieved in the first stage. The AMP-based classification method demonstrated a strong performance, particularly in the sinusoidal (class a) and sinc (class b) categories, where the AMPs exhibited distinctive and consistent variations. Although some overlap may occur between more complex classes, such as sinusoidal+sinc (Palmer series, class c) and constant (class d), the AMP-based method remains highly effective for initial radar signal differentiation.

This AMP-based classification method not only provides high classification accuracy but also lays the foundation for a more detailed classification in the second step, where a visibility-graph-based method is applied to classify ambiguous signals within each class.

#### 5.3.2. Stage 2: Visibility-Graph-Based Scan Pattern Classification

For radar reception signals with the 11 distinct radar scan pattern types, Stage 1, i.e., the AMP-based signal class classification in a tree structure, demonstrated excellent performance. Building on this, the classification performance of the proposed technique utilizing visibility graphs for detailed classification in the second stage was analyzed. These results were compared with those of a conventional approach that employs spectrograms for signal classification. Spectrograms, which are widely used in signal processing, convert time-domain signals into frequency components and offer a visual representation of the frequency distribution of the signal over time. Although this method is effective for classifying radar signals based on their spectral characteristics, it has notable limitations. This method incurs high computational costs because of the need for a high-resolution analysis, and the classification accuracy may degrade when the scan patterns exhibit high similarity.

In contrast, the proposed visibility-graph-based method incorporates both global and local patterns of time-series data, enabling more precise analysis of dynamic signal changes. This approach has significant potential for achieving a more efficient and accurate classification of radar signals. The bulleted lists show this situation.

Table 4, Table 5 and Table 6 summarize the performances of the GoogLeNet and ResNet models when classifying radar scan patterns across various classes using both the spectrogram- and visibility-graph-based methods. For example, in the classification of sinc scan patterns, such as circular, sector, helical, and raster scans, the visibility-graph-based methods consistently outperformed the spectrogram-based methods. In Table 4, the visibility-graph-based ResNet model shows accuracy rates of over 95%, with satisfactory performance in the sector and circular scan patterns, achieving F1-scores of 0.9215 and 0.9154, respectively. These results are significantly better than those of the spectrogram-based method, particularly in terms of precision and recall.

Similarly, in Table 5, which covers the Palmer scan patterns, the visibility-graph-based approach demonstrates superior classification performance across all categories. Using visibility graphs, the ResNet model yielded an accuracy of more than 96% for the Palmer circular and Palmer sector scans, with a high F1-score of 0.9314. The GoogLeNet model, although slightly lower in accuracy than ResNet, also benefitted from the visibility graph approach, with F1-scores above 0.9107 for all the Palmer classes.

Table 6 presents the classification results for switching class patterns, such as electronic lobe-switching and lobe-switching. Here, the visibility-graph-based method again demonstrated its strength, particularly with the ResNet model, achieving an accuracy of 96.7% for both classes and F1-scores of 0.9669 and 0.9664, respectively. In contrast, the spectrogram-based methods exhibited a lower classification performance, with F1-scores for electronic lobe-switching of approximately 0.8524 for GoogLeNet and 0.8624 for ResNet.

These results demonstrate that the proposed visibility-graph-based method provides significant improvements in the classification performance across all the radar scan patterns, particularly in distinguishing similar scan types. Additionally, advanced deep learning models, such as GoogLeNet and ResNet, can accurately and reliably classify complex radar signals, demonstrating the robustness of the visibility-graph-based scan pattern classification technique.

**Figure 10:**
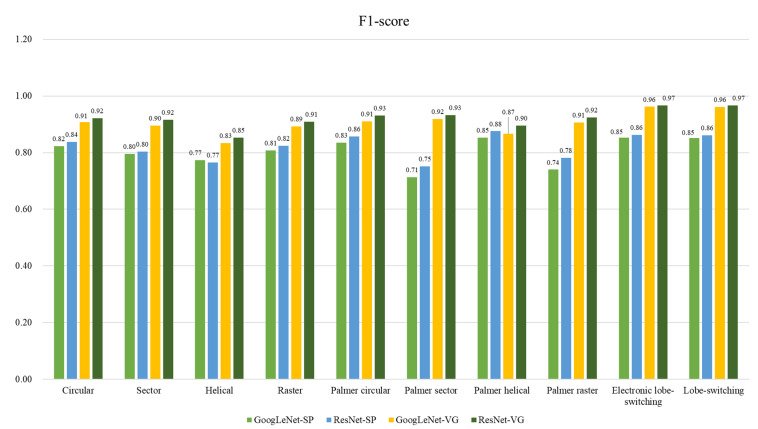
F1-score graph of scan pattern classification methods.

An F1-score graph is shown in Figure 10 to further illustrate the performance of the classification models in Stage 2. While the precise performance metrics are presented in Table 6, the graph provides a visual representation of the F1-scores for each radar scan pattern class across the different classification methods: GoogLeNet-SP, ResNet-SP, GoogLeNet-VG, and ResNet-VG. In the graph, the scores are rounded to two decimal places for simplicity, making it easier to identify the performance differences among the methods. Here, SP refers to spectrogram-based classification, and VG denotes the visibility-graph-based approach.

The results demonstrate that the visibility-graph-based methods consistently outperformed the spectrogram-based methods across nearly all the scan pattern classes. For instance, in the circular scan class, GoogLeNet-VG achieved an F1-score of 0.91, which is significantly higher than that (0.82) achieved by GoogLeNet-SP. This trend continued across more complex scan types, such as the Palmer circular and Palmer helical, where the visibility-graph-based methods showed evident improvements in the classification accuracy compared with the spectrogram-based approaches.

In addition, ResNet-VG generally performed slightly better than or equal to GoogLeNet-VG for most radar scan classes. This is particularly evident in the Palmer circular and Palmer sector classes, where ResNet-VG achieved F1-scores of 0.93 and 0.92, respectively, highlighting its robustness in handling more challenging signal classifications. Overall, the graph serves as a compelling visualization of how the visibility-graph-based methods enhance classification performance across different radar scan patterns, highlighting the advantage of these approaches over traditional spectrogram-based techniques.

To further validate the importance of feature extraction using AMP and VG, preliminary experiments were conducted by directly inputting raw radar signal data into deep learning classifiers, including GoogLeNet and ResNet. For single signals, classification accuracy dropped by 20–30%, resulting in an accuracy of approximately 60–70%. In more complex scenarios, particularly with Palmer series patterns or in multi-signal environments, accuracy decreased to around 40–50%. These results suggest that raw data alone do not effectively capture the critical amplitude and periodic variations necessary for distinguishing intricate radar patterns, especially in low-SNR and multi-signal environments. Therefore, structured preprocessing with AMP and VG is essential for achieving reliable classification accuracy in complex radar signal scenarios.

#### 5.3.3. Classification Accuracy Across Different SNR Levels

To evaluate the robustness of the proposed AMP + visibility graph (VG) framework, we compared its classification performance with traditional spectrogram-based methods across a range of SNR levels, from −10 dB to 20 dB. Four classification methods were tested, combining GoogLeNet and ResNet with spectrogram and visibility graph feature sets.

**Figure 11:**
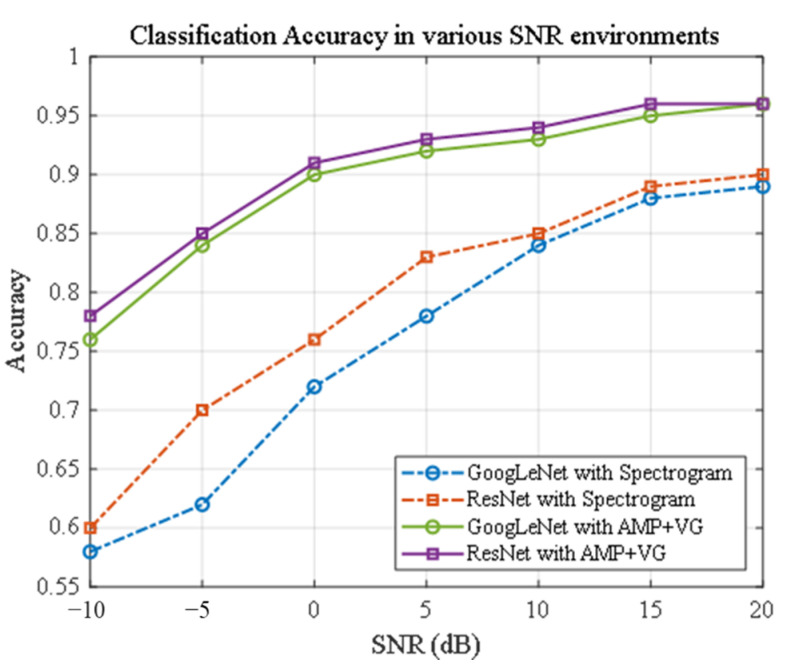
Comparison of classification accuracy in various SNR environments.

As illustrated in Figure 11, the AMP + VG-based methods consistently outperform the spectrogram-based methods across all SNR levels. Notably, at 0 dB, the AMP + VG approaches achieve an impressive accuracy of approximately 90%, demonstrating their robustness even in severely noisy environments. By 15 dB, the AMP + VG methods reach around 95% accuracy, compared to only about 88% for the spectrogram-based approaches, highlighting the effectiveness of the AMP + VG framework in preserving classification accuracy across challenging SNR conditions.

The enhanced performance of the AMP + VG approach can be attributed to its two-stage structure, which incorporates both amplitude-based and structural features. The first stage uses AMP analysis to categorize signals based on amplitude patterns, effectively isolating core signal characteristics and reducing background noise influence. In the second stage, the visibility graph method captures temporal and structural features within these categories, enabling precise classification of radar scan patterns.

### 5.4. Qualitative Comparison with Recent Radar Scan Pattern Classification Techniques

To complement the quantitative analysis in Section 5.3, Table 7 presents a qualitative comparison between the proposed AMP + visibility graph (VG) method and recent radar scan pattern classification techniques [4,11]. The proposed AMP + VG method demonstrates effectiveness in classifying complex radar patterns, such as those in low-SNR conditions and environments with overlapping signals. Unlike existing methods, which either exclude Palmer patterns or provide only broad grouping, our approach integrates AMP and VG techniques to achieve detailed classification, distinguishing specific sub-patterns within the Palmer series.

Furthermore, our method was tested for low-SNR effectiveness, utilizing AMP-based initial grouping to isolate noise and allowing the visibility graph analysis to focus on structural signal features. In contrast, the methods of Ayazgok et al. [4] and Kocamış et al. [11] have not been evaluated under low-SNR conditions. By employing a two-stage structure, our approach demonstrated improved robustness and adaptability, providing a versatile solution for radar scan pattern classification in challenging conditions, such as low-SNR environments and multi-signal overlap.

## 6. Conclusions

In this paper, we presented a two-stage radar signal classification framework that combines AMP-based initial categorization with a visibility-graph-based refinement process. The AMP analysis proved effective in separating radar signals into broad categories, such as conical, sectoral, and tracking patterns, providing a solid foundation for further classification. The introduction of visibility graphs in the second stage facilitated a more detailed analysis of the temporal relationships within the signals, significantly improving the classification accuracy for complex patterns, particularly those found in the Palmer series. Our experimental results demonstrated that the proposed method consistently outperformed traditional spectrogram-based approaches, particularly under low-SNR conditions and in cases involving radar signals with overlapping features. Furthermore, the qualitative comparison with recent classification techniques highlighted the adaptability and effectiveness of our approach in addressing limitations commonly encountered in complex radar environments. The integration of advanced deep learning models, namely, GoogLeNet and ResNet, further enhanced the robustness and reliability of our classification algorithm, contributing to its versatility in real-world scenarios. This study provides a promising framework for the real-time classification of radar signals, offering significant advancements for applications in EW and other radar-based systems. In the future, we plan to extract additional feature vectors from the visibility graph obtained from the scan pattern dataset of the actual signals received through scanning radars. We will compare and analyze the performances of various deep-learning-based image classification models under different operational conditions. These results are expected to contribute to the development of advanced radar reception signal analysis algorithms, especially for complex EW simulation environments.

## Figures and Tables

**Table 1:** Classes and their corresponding scan types.

Class	Label	Corresponding Scan Type
Sinusoidal	a	Conical
Sinc	b	Circular, sector, raster, helical
Sinusoidal+Sinc	c	Palmer circular, Palmer sector, Palmer raster, Palmer helical
Constant	d	Electronic lobe-switching, lobe-switching

**Table 2:** Simulation parameters for the training and test datasets.

Parameters	Value/Intervals
Image size	600 × 600
The number of images	2600 (training)/700 (test)
The number of radar signal per image	{1, 2, 3, 4, 5}
Sinusoidal peak value	2–5 dB
The number of lobes	{2, 4}
PRI modulation type	constant
PRI value	100–1000 μs

**Table 3:** Classification accuracy of radar scan types using AMP-based signal categorization.

Class	Corresponding Scan Type	Accuracy (%)
Sinusoidal	Conical	97.1
Sinc	Circular, sector, raster, helical	95.3
Sinusoidal+Sinc	Palmer circular, Palmer sector, Palmer raster, Palmer helical	93.4
Constant	Electronic lobe-switching, lobe-switching	96.7

**Table 4:** Performance of the sinc class scan pattern classification.

Classification Methods		Circular	Sector	Helical	Raster
GoogLeNet using spectrogram	Accuracy (%)	91.0	89.7	89.2	90.2
	Precision (%)	84.4	79.8	74.0	82.0
	Recall (%)	80.3	79.2	81.1	79.7
	F1-score	0.8228	0.7950	0.7736	0.8082
ResNet using spectrogram	Accuracy (%)	91.8	90.2	88.5	91.1
	Precision (%)	84.1	80.3	75.4	83.4
	Recall (%)	83.3	80.5	77.7	81.4
	F1-score	0.8372	0.8041	0.7654	0.8243
GoogLeNet using visibility graph	Accuracy (%)	95.3	94.8	91.6	94.6
	Precision (%)	90.8	88.8	83.9	89.3
	Recall (%)	90.6	90.4	82.8	89.1
	F1-score	0.907	0.8958	0.8335	0.8921
ResNet using visibility graph	Accuracy (%)	96.1	95.8	92.6	95.4
	Precision (%)	92.6	90.8	85.6	90.9
	Recall (%)	91.7	92.3	85.0	90.8
	F1-score	0.9215	0.9154	0.8526	0.9085

**Table 5:** Performance of the sinusoidal+sinc (Palmer) class scan pattern classification.

Classification Methods		Palmer Circular	Palmer Sector	Palmer Helical	Palmer Raster
GoogLeNet using spectrogram	Accuracy (%)	91.7	85.7	92.6	87.0
	Precision (%)	83.5	71.4	85.3	74.0
	Recall (%)	83.5	71.4	85.3	74.0
	F1-score	0.8345	0.7136	0.8529	0.7399
ResNet using spectrogram	Accuracy (%)	92.9	87.6	93.8	89.0
	Precision (%)	85.1	75.4	87.6	78.4
	Recall (%)	86.3	75.0	87.5	77.8
	F1-score	0.8570	0.7517	0.8755	0.7813
GoogLeNet using visibility graph	Accuracy (%)	95.5	96.0	93.3	95.3
	Precision (%)	91.3	90.5	87.7	90.8
	Recall (%)	90.9	93.3	85.7	90.5
	F1-score	0.9107	0.9189	0.8670	0.9067
ResNet using visibility graph	Accuracy (%)	96.6	96.7	94.7	96.2
	Precision (%)	93.4	92.4	90.0	92.6
	Recall (%)	92.9	94.2	89.1	92.4
	F1-score	0.9314	0.9327	0.8953	0.9246

**Table 6:** Performance of the constant (switching) class scan pattern classification.

Classification Methods		Electronic Lobe-Switching	Lobe-Switching
GoogLeNet using spectrogram	Accuracy (%)	85.2	85.2
	Precision (%)	85.7	84.7
	Recall (%)	84.8	85.5
	F1-score	0.8524	0.8509
ResNet using spectrogram	Accuracy (%)	86.2	86.2
	Precision (%)	86.7	85.7
	Recall (%)	85.8	86.5
	F1-score	0.8624	0.8610
GoogLeNet using visibility graph	Accuracy (%)	96.2	96.2
	Precision (%)	96.7	95.7
	Recall (%)	95.7	96.6
	F1-score	0.9619	0.9615
ResNet using visibility graph	Accuracy (%)	96.7	96.7
	Precision (%)	97.3	96.0
	Recall (%)	96.1	97.3
	F1-score	0.9669	0.9664

**Table 7:** Qualitative comparison of the proposed method with recent classification techniques.

Criteria	Ayazgok et al. [4]	Kocamış et al. [11]	Proposed Method
Classification feature	Beam characteristics	AMP	AMP+Visibility Graph
Range of patterns	7 patterns (Palmer series excluded)	Limited (No Palmer)	11 patterns (Palmer series included)
Multi-signal environments	Restricted	Moderate	Reliable
Low-SNR effectiveness	Limited (tested at 15–25 dB)	Not tested	Tested (−10 to 20 dB)
Complex pattern Adaptability	Not tested	Not tested	Tested
Levels of classification detail	Classification of 7 distinct patterns	Broad grouping only	Detailed classification (like Palmer series sub-patterns)

## Data Availability

The methods for generating the data are described within the article.
